# Dr. Robinson *Versus* Dr. Younger, in Reference to Implantation

**Published:** 1897-06

**Authors:** Louis Jack


### DR. ROBINSON *VERSUS* DR. YOUNGER, IN REFERENCE TO IMPLANTATION.

BY LOUIS JACK, D.D.S.

In the paper read by me before the Academy of Stomatology of Philadelphia, December, 1895, upon plantation of teeth, and published in the INTERNATIONAL DENTAL JOURNAL, March, 1896, it was stated that implantation of teeth undoubtedly originated with Dr. Younger, giving the date of his first operation of this nature as June, 1885. Thereupon, a disclaimer to this credit was made to me by Dr. M. H. Robinson, of Alameda, California, who states that he first described the operation of inserting teeth in the alveolus where teeth had long been absent. As evidence of this he cites a paper read by him before the California Dental Association in 1880, and published in the *Dental Jairus.* Further, he declared that before the same Association, in 1881, he defended the propriety of the same operation, he stating that Dr. Younger was present.

Dr. Robinson in his communication required me in the cause of historical accuracy to correct my apparent misstatement. This I can only do by giving a short abstract of the proceedings of the California Dental Association, as the amount of matter bearing on the question is too great to be copied *verbatim.* It should here be stated with regret that the stenographic notes of the discussions, particularly of the year 1881, have been destroyed.

In the *Dental Jairus*, volume i., No. 8, page 339, August, 1880, Dr. Robinson described substantially, in the following quotation, the operation since designated as implantation. In this article the feasibility of inserting teeth in vacant positions of the alveolus is strenuously advised, and the operation is confidently laid down as correct treatment. It does not, however, appear that at this time Dr. Robinson had put in actual practice the operation he advised.

"The almost universal success of these operations—*i.e.*, transplantations—is astonishing when we look at the records of them and see that the way they are performed is not only careless, but in many cases in utter violation of every principle that should guide us in such operations. When the genius of the profession is directed to these operations, so as to give them the attention they merit, we will soon know the modes and principles so well that transplanting or replanting will be operations with fewer failures than now follow ordinary nerve-devitalization. *Furthermore, we will soon see teeth transplanted into alveoli that have been without teeth for years.* Let me make this point emphatic. Mr. A. has lost an incisor and wears a cumbrous plate. He presents himself to the dentist and says he wants a real tooth there instead of that cumbrous plate-tooth arrangement. The dentist says call at such a time and I will perform the operation. In the mean time the dentist has found a suitable tooth for transplanting into Mr. A.'s mouth. He calls; *the dentist makes a socket in the alveolus, inserts the tooth*, and if a real live, useful tooth is a desirable success, then transplanting is a success, for it gives that result. So confident am I that teeth can be successfully *planted* into an alveolus where the teeth have been out for years, in a socket prepared by the dentist to receive them, that I would have no fears or hesitation in performing it whenever proper opportunity occurred."

On June 15, 1881, before the California State Dental Association, Dr. Younger reports since the meeting of 1880 three more cases of transplantation. "In one of these cases the root was *mutilated* on the whole of the lingual and buccal surfaces to adapt it to the socket into which it was inserted."

In one other of the cases he states he will have "either to deepen the socket by drilling into the bone or by pressure with the old root to induce absorption and thus extend the cavity." Here Dr. Younger was awaiting the offering of a suitable tooth, meanwhile keeping the socket open by the application of the previous diseased tooth. (See the Transactions for 1881.)

In the Transactions for 1883, page 277, appears the report concerning the altered root which had continued serviceable for nearly two years.

*Page 267, same Session.*—Dr. Younger describes his intention to remove two bicuspids for a boy and to insert them in vacancies in the mother's mouth where the teeth had been absent for five or six years.

*Page 269, Ibid.*—Dr. Younger disclaims having used teeth which had long been extracted (*i.e.*, old teeth), he stating that the only teeth he had utilized in this way were two which he had kept in condition for use by inserting them in a cock's comb.

*Transactions Sixteenth Annual Session, August, 1885.*— Dr. Younger reports a case of drilling a cavity in the alveolar process from which a root had been extracted four years before, and in which artificial socket he transplanted a healthy tooth.

*Transactions of the California State Dental Association, Seventeenth Session, July, 1886, Page 436.*—"*Dr. Younger.*—Transplantation and implantation, as I call my new operation, has so far realized my most sanguine expectations."

*Ibid.*—Dr. Younger reports a case where, after a tooth had been extracted for over thirteen months, he formed a new alveolus and inserted it in its former place; "union took place as rapidly and as thoroughly as if it had been a fresh tooth."

*Ibid., Page 451.*—"Dr. Younger gave a clinic on implantation,—transplanting a tooth into an artificial socket drilled into the alveolar process of Dr. J. E. Cummings."

*Ibid Page 498.*—Dr. Younger was questioned, "How long does the operation of implantation extend back?"

"*Dr. Younger.*—To the 15th of June of last year (1885)."

I have given such an abstract of the essential points from the proceedings of the California State Dental Association as will enable the profession to judge how much credit to extend to Dr. Robinson and how far Dr. Younger may be indebted to the outline of Dr. Robinson published in 1880, as suggesting to him the operation of implantation.

